# Supplementary material for: Assessment of the technological viability of photoelectrochemical devices for oxygen and fuel production on Moon and Mars
Source: Nat Commun. 2023 Jun 6;14:3141. doi: 10.1038/s41467-023-38676-2 (PMC10244351; doi:10.1038/s41467-023-38676-2)
Supplement: Supplementary file 1 — Supplementary Information [file 41467_2023_38676_MOESM1_ESM.pdf]

Assessment of the Technological Viability of Photoelectrochemical Devices for Oxygen and Fuel Production on Moon and Mars

Supporting Information (SI)

Byron Ross[1], Sophia Haussener[2], Katharina Brinkert*[1,3]

[1]*Department of Chemistry, University of Warwick, Coventry, CV4 7AL, UK*
[2] *Institute of Mechanical Engineering, Ecole Polytechnique Fédérale de Lausanne (EPFL), 1015 Lausanne, Switzerland*
[3]*ZARM - Center for Applied Space Technology and Microgravity, University of Bremen, 28359 Bremen, Germany*

Content

	Page
**I PEC Model**	**2**
SI Table 1	5
**II GDE - Analytical Model**	**6**
SI Table 2	9
**III Radiative Transfer Model**	**10**
SI Figure 1	11
SI Figure 2	12
**IV Model Validations**	**13**
SI Table 3	13
**V Solar Fuel Production Model**	**14**
SI Figure 3	15
**VI H-Cell-type CO_2R and COR Solubility-Limited Current Density**	**16**
SI Table 4 and SI Figure 4	16
**VII GDE-PV Design**	**17**
SI Table 5 and SI Figure 5	17
SI Figure 6	18
**VIII Lunar and Martian Soil Dust Performance Analysis**	**19**
SI Figure 7	19
SI Figure 8 and SI Table 6	20
SI Table 7 and SI Figure 9	21
**IX Natural Resource Abundances**	**22**
SI Table 8	22
SI Table 9	23
**X References**	**24**

I PEC Model

We assume that all device junctions are optically connected in series and are to be constructed with descending bandgap (ε_g) values from top to bottom. The photogenerated current (i_{ph}) at each junction, and subsequently the device limited current (i_{lim}), are given by:

$$i_{ph_{(T)}} = q \cdot \int_{\varepsilon_g(T)}^{\infty} \phi(\lambda) \cdot IPCE \cdot d\lambda \tag{1}$$

$$i_{ph_{(M)}} = q \cdot \int_{\varepsilon_g(M)}^{\varepsilon_g(T)} \phi(\lambda) \cdot IPCE \cdot d\lambda \tag{2}$$

$$i_{ph_{(B)}} = q \cdot \int_{\varepsilon_g(B)}^{\varepsilon_g(M)} \phi(\lambda) \cdot IPCE \cdot d\lambda \tag{3}$$

$$i_{lim} = min\left\{i_{ph_{(T)}}, i_{ph_{(M)}}, i_{ph_{(B)}}\right\} \tag{4}$$

Here, ϕ is the incident spectral photon flux, which describes the energetic distribution of photons reaching a given planetary surface, q is the elementary charge constant, and $IPCE$ is the incoming photon conversion efficiency that reflects non-ideal photon absorption at each junction. To calculate the minority carrier flow (i_θ) in the neutral space charge region or the so-called 'reverse saturation current', a detailed balance model inaugural in Shockley and Queisser's single-junction photovoltaic efficiency article is employed[1]:

$$i_{\theta_{(t)}} = \left\{\frac{q}{4\pi^2\hbar^3 \cdot c^2 \cdot \eta^{ext}}\right\} \int_{\varepsilon_g(T)}^{\infty} \frac{\varepsilon^2}{e^{\left(\frac{\varepsilon}{k_b T}\right)} - 1} \cdot d\lambda \tag{5}$$

$$i_{\theta_{(m)}} = \left\{\frac{q}{4\pi^2\hbar^3 \cdot c^2 \cdot \eta^{ext}}\right\} \int_{\varepsilon_g(M)}^{\varepsilon_g(T)} \frac{\varepsilon^2}{e^{\left(\frac{\varepsilon}{k_b T}\right)} - 1} \cdot d\lambda \tag{6}$$

$$i_{\theta_{(b)}} = \left\{\frac{q}{4\pi^2\hbar^3 \cdot c^2 \cdot \eta^{ext}}\right\} \int_{\varepsilon_g(B)}^{\varepsilon_g(M)} \frac{\varepsilon^2}{e^{\left(\frac{\varepsilon}{k_b T}\right)} - 1} \cdot d\lambda \tag{7}$$

$$\eta^{ext} = \frac{e^{\left(\frac{q \cdot V_{oc}}{k \cdot T}\right)} \cdot i_\theta}{i_{sc}} \tag{8}$$

The external radiative efficiency (η^{ext}) quantifies the rate of non-radiative recombination losses[2-4]. h is Planck's constant, k_b is Boltzmann's constant, T is the temperature, and c the speed of light. The open circuit voltage of each PEC junction ($V_{oc\,(x)}$) - or the maximum available individual junctional potential - is found through re-arrangement of the ideal diode equation assuming zero net current.

$$V_{oc\,(T)} = \frac{z_D k_b T}{q} ln\left(\frac{i_{lim}}{i_{\theta\,(T)}} + 1\right) \tag{9}$$

$$V_{oc\,(M)} = \frac{z_D k_b T}{q} ln\left(\frac{i_{lim}}{i_{\theta\,(M)}} + 1\right) \tag{10}$$

$$V_{oc\,(B)} = \frac{z_D k_b T}{q} ln\left(\frac{i_{lim}}{i_{\theta\,(B)}} + 1\right) \tag{11}$$

$$V_{PEC} = \sum_{i=T}^{M,B} V_{oc} \tag{12}$$

The maximum achievable voltage across the PEC system (V_{PEC}) is then given by the sum of junction potentials, where the diode ideality factor (z_D) is taken to be 1. We modify the ideal diode equation[5] with series (R_s) and shunt (R_{sh}) resistance terms resulting in a transcendental equation which is solved iteratively in MATLAB using fsolve:

$$i_{PEC} = i_{ph} - i_\theta e^{\left(\frac{q(V_{PEC}+i_{PEC}R_s)}{z_D k_b T}\right)} - \frac{V_{PEC} + i_{PEC}R_s}{R_{sh}} \tag{13}$$

We employ the inverse-hyperbolic sign formulation of the Butler-Volmer equation[2] to account for PEC electrocatalytic overpotentials ($\eta_{(cat)}$) arising from electrocatalyst electron transfer kinetics:

$$\eta_{(PA)} = \frac{RT}{\alpha F n_{e^-}} sinh^{-1}\left(\frac{i_{PEC}}{2 \cdot i_{\theta\,PA}}\right) \tag{14}$$

$$\eta_{(PC)} = \frac{RT}{\alpha F n_{e^-}} sinh^{-1}\left(\frac{i}{2 \cdot i_{\theta\,PC}}\right) \tag{15}$$

$$\eta_{(cat)} = \left|\eta_{(cat,a)}\right| + \left|\eta_{(cat,c)}\right| \tag{16}$$

$\eta_{(cat,a)}$ and $\eta_{(cat,c)}$ are the electrocatalytic overpotentials associated with the photoanode and photocathode, respectively, the charge transfer coefficient (α) describes the transition state symmetry, and $i_{\theta\,PA}$ and $i_{\theta\,PC}$ are the catalytic exchange current densities values for the photoanode and photocathode, respectively, which denote the overall ability of our electrocatalyst to effectively transfer electrons. The electrochemical potential requirement (U_θ) for each PEC reaction to proceed can be described as[6]:

$$U_\theta = U_{\theta_{ox}} - U_{\theta_{red}} \tag{17}$$

Where $U_{\theta_{ox}}$ and $U_{\theta_{red}}$ are the oxidative and reductive half-cell potentials, respectively. The device efficiency ($\eta_{(PEC)}$) can now be calculated as follows:

$$\eta_{(PEC)} = \frac{i_{op}(V_{op}) \cdot U_\theta \cdot FE(\%)}{P_{in}} \tag{18}$$

Here, i_{op} is the operating current - the point of maximum efficiency - when the current density is maximised. It occurs when the device voltage equals the overall required electrochemical potential ($U_{\theta_{PEC}}$). Here:

$$V_{op}(i_{op}) = U_{\theta_{PEC}} = U_\theta + |\eta_{(cat,a)}| + |\eta_{(cat,c)}| + iR_{series} \tag{19}$$

P_{in} is the integrated power density incident on the photoabsorber taken as 1000 Wm^{-2} and 1367 Wm^{-2} for AM 1.5 G and AM 0, respectively. For MAM 1.5, we obtain 369 Wm^{-2} from integrating the spectral flux across 280 - 4000 nm. Given a known operating voltage ($V_{op}(i_{op})$), the corresponding operating current can be calculated with SI equation (13). The Faradaic efficiency (FE) is the ratio of the desired product yield which can experimentally be calculated as follows[7]:

$$FE(\%) = \frac{F \cdot \phi_{gas} \cdot F_m \cdot n_{e^-}}{i_{FE}} \tag{20}$$

Here, ϕ_{gas} is volume fraction of gases, F_m equates to the anolyte molar gas flow rate, and i_{FE} is the measured current. All realistic model values are reported having been multiplied by the appropriate Faradaic efficiency (SI Table 1), thermodynamic limiting models are assumed to have 100% Faradaic efficiency. The device efficiency calculations can now be run over several thousand possible bandgap combination iterations, depending on computational resources. Generally, there is an exponential increase in computational resources with each subsequent junction being modelled. Once a relative maximum efficiency is found, the predicted bandgap combinations can be fed back into the ideal diode equation to simulate the current-voltage characteristics and gauge the current densities of the modelled scenario.

Parameters	WS (HER)	Ref	WS (OER)	Ref	Unit
Electrolyte	0.5 M H_2SO_4	8	1 M H_2SO_4	9	M
Electrocatalyst	Pt	10	[4]$Ru_{0.95}Ir_{0.05}O_2$	11	-
i_θ	[1]9.8	10	$6.8 \cdot 10^{-2}$	11	A m^{-2}
α	[2]0.209	12	[5]0.835	12	-
FE_{PC}	[3]0.83	8	[4]0.93	9	-
U	0	-	1.229	-	V
$IPCE$	0.90			2	-
η^{ext}	3%			2	-
R_s	0.0015			-	Ω m^{-2}
R_{sh}	0.4			-	Ω m^{-2}
T	298.15	-	K		

SI Table 1 | Summary of state-of-the-art device parameters used in water-splitting PEC models. [1]Pt(110), 0.05 M H_2SO_4 . [2]20°C. [3]Pt/C estimated from literature Fig. 5g. [4]0.5 M H_2SO_4. [5]20°C.

II GDE - Analytical Model

The GDE (gas diffusion electrode) model was adapted and extended from previous literature[13] in which the authors give an extensive derivation of their original model. We outline the main body of equations implemented in MATLAB to extend this model to CH_4 production and implement this for unassisted PV-driven CO_2R devices on Earth and Mars. Two separate devices were designed for CO and CH_4 production. Then, a PV was designed for each device to perform the unassisted CO_2R under different irradiance conditions, using the aforementioned equations in the PEC model description.

Initial carbonate equilibrium

$$K_1: CO_2 + OH^- \overset{k_1}{\Leftrightarrow} HCO_3^- \qquad (21)$$

$$K_2: HCO_3^- + OH^- \overset{k_2}{\Leftrightarrow} H_2O + CO_3^{2-} \qquad (22)$$

$$K_3: CO_2 + H_2O \overset{k_3}{\Leftrightarrow} HCO_3^- + H^+ \qquad (23)$$

$$K_4: HCO_3^- \overset{k_4}{\Leftrightarrow} CO_3^{2-} + H^+ \qquad (24)$$

$$K_w: H_2O \overset{k_w}{\Leftrightarrow} H^+ + OH^- \qquad (25)$$

DIC electrolyte solution

The dissolved inorganic carbon (DIC) refers to the total contribution of CO_2, HCO_3^-, and CO_3^{2-} in solution. The following equation was solved iteratively using fsolve in MATLAB to calculate the concentration of protons [H^+] and subsequently, the pH.

$$pH = -log_{10}\left(\frac{[H^+] - \left([H^+]^4 + (k_1 + [KHCO_3]) \cdot [H^+]^3 + (k_1 \cdot k_2 - k_w) \cdot [H^+]^2 + \left(-k_1 \cdot (k_w + [KHCO_3]/k_2)\right) \cdot [H^+]\right)}{4[H^+]^3 + 3(k_1 + [KHCO_3]) \cdot [H^+]^2 + 2(k_1 \cdot k_2 - k_w)[H^+]}\right) \qquad (26)$$

The concentration of each species in the electrolyte can now be expressed as:

$$[CO_2] = \frac{[KHCO_3]}{\left(\frac{k_1}{10^{-pH}}\right) + k_1 \cdot \left(\frac{k_2}{(10^{-pH})^2}\right) + 1} \qquad (27)$$

$$[HCO_3^-] = \frac{[CO_2] \cdot k_1}{10^{-pH}} \qquad (28)$$

$$[CO_3^{2-}] = \frac{[CO_2] \cdot k_1 \cdot k_2}{(10^{-pH})^2} \qquad (29)$$

$$[OH^-] = \frac{k_w}{10^{-pH}} \qquad (30)$$

Flow channel characteristics

The flow channel characteristics describe the distribution of fluid within the model domain. We assume no dispersed phase and the bulk electrolyte pH is not perturbed by electrode reactions.

$$Re = \frac{\rho v L}{\mu} \tag{31}$$

Here, Re is the Reynolds number, ρ is the fluid density, v is the flow velocity, L the linear dimension, and μ the dynamic viscosity. The average developing boundary layer thickness (δ^i) for each species is then given by:

$$\delta^i = 1.607 \frac{3}{4} \sqrt[3]{\frac{W_{channel} \cdot D^i \cdot L_{channel}}{v}} \tag{32}$$

$W_{channel}$ is the flow channel width, $L_{channel}$ is the flow channel length, and D^i is the diffusion coefficient of species i. This allows calculation of the mass transfer coefficient at the electrolyte flow channel boundary for each species (K_{EL}^i).

$$K_{EL}^i = \frac{D^i}{\delta^i{}_{eff}} \tag{33}$$

Transport

Transport is governed by the Nernst-Planck equation which can be simplified by assuming averaged ion concentrations, and zero flux at the electrolyte boundary for CO_2[13]. z^i is the ionic charge, ϵ is the catalyst porosity, and a is the catalyst layer volumetric surface area. The governing conservation equation is then formulated as:

$$-D^i \frac{\partial c^i}{\partial x} = \frac{D^i z^i F c^i}{RT} \frac{\partial \phi}{\partial x} \tag{34}$$

The Bruggeman correction, where D^i is the diffusivity, is given by:

$$D^i = D_0^i \cdot \epsilon_{CL}{}^{3/2} \tag{35}$$

This can be solved linearly though the Thiele modulus:

$$M_0 = \sqrt{\frac{kL^2}{D^0}} \tag{36}$$

The process for obtaining the corrected [CO_2] is initiated by calculating the [CO_2] excluding ionic effects, the equilibrium reactions, loss of CO_2 in the electrolyte channel, and the Nernst correction[13]. This initially calculated [CO_2] value is then fed into the expression for [OH^-] below in order to determine the OH^- and HER rates of production[13]. This process is then repeated to update [CO_2] considering equilibrium reactions[13].

$$k_0 = \frac{a}{n_{e^-}F} \frac{i^*_{CO_2R}}{[CO_2]_{ref}} exp\left(\frac{-\eta_{CO_2R}}{b_{CO_2R}}\right) \tag{37}$$

$$[CO_2] = \mathcal{H}_0 p^0 \frac{K^0_{DL} \cdot tanh(M_0)}{K^0_{DL} \cdot M_0 + k_0 \cdot L \cdot tanh(M_0)} = \mathcal{H}_0 p^0 \varepsilon^{tot}_0 \tag{38}$$

$$R^1_{H_2} = \frac{a \cdot i^*_{base}}{F} exp\left(\frac{-\eta_{base}}{b_{base}}\right) \tag{39}$$

$$[OH^-]_{EL} = [OH^-] + \frac{K^1_{EL} \cdot c^1_{EL} - J^3_{EL,0} + L \cdot R^1_{E,0}}{K^1_{EL} + n_{e^-}\epsilon \cdot L \cdot \underset{\rightarrow}{K^1} \cdot [CO_2]} \tag{40}$$

$\mathcal{H}_0$ is Henry's constant, p^0 is the partial pressure of CO_2, and K^0_{DL} is the gas diffusion layer mass transfer coefficient. The $[CO_3^{2-}]$ is then solved through the Lambert-W function:

$$[CO_3^{2-}] = A + \frac{1}{C} W(BCe^{-AC}) \approx A + \frac{1}{C} ln\left(\frac{BCe^{-AC}}{1 + ln\sqrt{BCe^{-AC}}}\right) \tag{41}$$

Where:

$$A = \frac{K^1_{EL} \cdot c^1_{EL} + n_{e^-}K^2_{EL} \cdot c^2_{EL} + K^3_{EL} \cdot c^3_{EL} + L \cdot R^1_{H_2} - K^1_{EL} \cdot [OH^-]}{n_{e^-}K^2_{EL}} \tag{42}$$

$$B = \frac{L \cdot R^1_{CO,1}}{n_{e^-}K^2_{EL}} exp\big(-[OH^-](h_{OH^-} + h_{K^+})\big) \tag{43}$$

$$C = h_{CO_3^{2-}} + n_{e^-} \cdot h_{K^+} \tag{44}$$

The corrected $[CO_2]$ is now given below, which considers salting-out:

$$[CO_2] \ = \mathcal{E}^{tot}_1 \cdot \mathcal{H}_0 \cdot p^0 exp\left(-[OH^-] \cdot h_{OH^-} - [CO_3^{2-}] \cdot h_{CO_3^{2-}} - ([OH^-] + n_{e^-}[CO_3^{2-}])h_{K^+}\right) \tag{45}$$

Electrode reaction kinetics

The electrode reaction kinetics are - similarly to the PEC model - described by the Butler-Volmer equation, but corrected for each given reaction pathway. The partial current density and electrode reaction potential for HER and CO_2R are then given by:

$$i_{(H_2OR)} = i_{\theta(H_2OR)} \cdot exp\left(\frac{-\eta^*_{H_2OR} \cdot F \cdot \alpha_{H_2OR}}{RT}\right) \tag{46}$$

$$i_{(CO_2R)} = \frac{[CO_2] \cdot i_{\theta(CO_2R)}}{[CO_2]_{ref}} exp\left(\frac{-\eta^*_{CO_2R} \cdot F \cdot \alpha_{CO_2R}}{RT}\right) \tag{47}$$

The Nernst corrected cathodic potential is given by:

$$\eta^*_{CO_2R} = V_{applied} - U_{CO_2R} + \frac{RT}{F}\ln\left(\frac{[OH^-]}{[OH^-]_{EL}}\right) \tag{48}$$

Parameters	CO_2RR (CO)	Ref.	CO_2RR (CH_4)	Ref.	Unit
Electrolyte	1 $KHCO_3$	13	1 $KHCO_3$	14	M
Electrocatalyst	Ag	13	Cu	14	-
$V_{applied}$	-1.48	Fitted	-1.64	Fitted	V
$i_{\theta_{CO_2R}}$	$4.71 \cdot 10^{-3}$	13	$1.41 \cdot 10^{-7}$	Fitted	A m^{-2}
α_{CO_2R}	0.44	13, 15	0.53	Fitted	-
$i_{\theta_{HER}}$	$1.16 \cdot 10^{-5}$	13	$6.09 \cdot 10^{-4}$	Fitted	A m^{-2}
α_{HER}	0.36	13, 15	0.29	Fitted	-
n_{e^-}	2	-	8	-	eV
U_{red}	-0.10	16	+0.17	16	V
$\epsilon_{catalyst}$	50%	13	60%	14	-
q_{flow}	0.50	13	90	14	ml min^{-1}
$L_{catalyst}$	$3.81 \cdot 10^{-6}$	13	$2.00 \cdot 10^{-7}$	14	m
$w_{channel}$	$1.50 \cdot 10^{-3}$	13, 17	$6.00 \cdot 10^{-3}$	Optimised	m
$L_{channel}$	0.02	13, 17	0.1	Optimised	m
$H_{channel}$	$5.00 \cdot 10^{-3}$	13, 17	$8.00 \cdot 10^{-3}$	Optimised	m
P_{CO_2}	1	13	0.6	14	atm
U_{HER}	0			16	V
D_{CO_2}	$1.91 \cdot 10^{-9}$			13, 18	m^2 s^{-1}
D_{OH}	$5.30 \cdot 10^{-9}$			13, 18	m^2 s^{-1}
D_{CO_3}	$1.18 \cdot 10^{-9}$			13, 18	m^2 s^{-1}
D_{HCO_3}	$0.92 \cdot 10^{-9}$			13, 18	m^2 s^{-1}
D_H	$9.31 \cdot 10^{-9}$			13, 18	m^2 s^{-1}
D_{H_2}	$4.50 \cdot 10^{-9}$			13, 18	m^2 s^{-1}
T	298.15			-	K
$i_{\theta_{OER}}$	$2.50 \cdot 10^{-5}$			19	A m^{-2}
α_{OER}	0.50			-	-
$\mathcal{H}_0$	34.06			13	mol m^{-3}
h_{OH^-}	$6.67 \cdot 10^{-5}$			13,20	m^3 mol^{-1}
$h_{CO_3^{2-}}$	$1.25 \cdot 10^{-4}$			13,20	m^3 mol^{-1}
h_{K^+}	$7.50 \cdot 10^{-5}$			13,20	m^3 mol^{-1}

SI Table 2 | Summary of state-of-the-art literature parameters used for our CO_2R models. The values for i_θ and α were found by empirically fitting experimental data[14] to the reported experimental conditions for the CH_4 model. $\epsilon_{catalyst}$ is the catalyst porosity, q_{flow} is the electrolyte flow rate, $L_{catalyst}$ is the catalyst later length, P_{CO_2} is the CO_2 input stream partial pressure, D_x is the diffusion coefficient for species x, $\mathcal{H}_0$ is Henry's constant, and h_x is the CO_2-ion salting out constant for ion x.

III Radiative Transfer Model

Given that radiation travels through the vacuum of space freely without being encumbered by energy losses[21], the solar irradiance spectrum reaching the top of the atmosphere (TOA) of an extra-terrestrial body at a given wavelength range is defined by the product of Earth's TOA spectrum and the ratio of the extra-terrestrial and Earth's integrated solar flux:

$$\phi_{TOA} = \left\{ \frac{\frac{R^2{}_{Sun}}{D^2{}_{ET}} \cdot \varepsilon\sigma T^4{}_{sun}}{\int_{\lambda_1}^{\lambda_2} \phi_{AM.0}} \right\} \cdot \phi_{AM.0} \tag{49}$$

Here, R_{Sun} is the radius of the sun, D_{ET} is the distance from the sun to the extra-terrestrial body of interest, T_{Sun} the temperature of the Sun's photosphere taken as 5800K, σ is the Stefan-Boltzmann constant, and ε the emissivity (taken to be 1). For characterisation of the solar flux reaching the surface of Mars, we employ radiative transfer theory, where the total intensity field I is described by the combination of direct I^{dir} and diffuse beam I^{scat} components[22]:

$$I(z, \mu, \Phi) = I^{dir}(z, \mu_0, \Phi_0) + I^{scat}(z, \mu, \Phi) \tag{50}$$

Here, z corresponds to the altitude, μ is $cos\theta$, Φ is the azimuth angle, and μ_0 is the solar zenith angle. We chose the pseudo-spherical approximation of the radiative transfer equation, where the direct beam component is described as[22]:

$$I^{dir}(r, \mu) = I^0 e^{-ch(r, \mu_0)} \tag{51}$$

The Chapman function (ch) defines the extinction path in a spherical atmosphere, the corresponding diffuse beam component is found through[22]:

$$\begin{aligned} -\mu \frac{dI(\tau, \mu, \Phi)}{d\tau} = I(\tau, \mu, \Phi) - \frac{\omega(r)}{4\pi} \int_0^{2\pi} d\Phi' \int_{-1}^{1} d\mu' p(\tau, \mu, \Phi; \mu', \Phi') I(\tau, \mu', \Phi) \\ - \left(1 - \omega(\tau)\right) B[T(\tau)] - \frac{\omega(\tau) I^0}{4\pi} p(\tau, \mu, \Phi; \mu_0, \Phi_0) e^{-ch(r, \mu_0)} \end{aligned} \tag{52}$$

Here, the $B[T(\tau)]$ is the Planck function, τ is the optical depth of the medium and ω is single-scattering albedo[22].

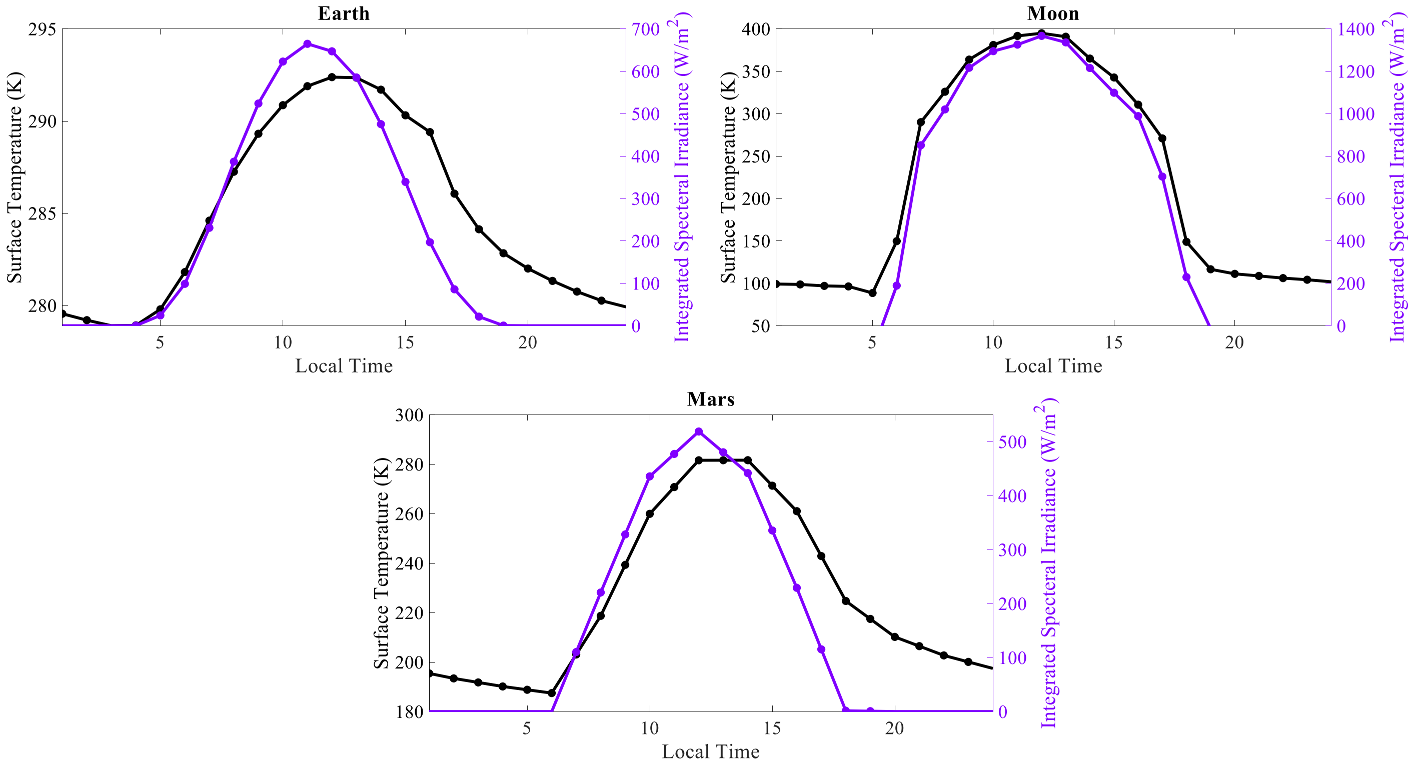

SI Figure 1 | Solar irradiance spectrum and celestial irradiance and temperature cycles. Local time for Earth and Mars corresponds to 1/24 of a full local day, while for the Moon this equals to 1/24 of a Lunar month. Earth's cycle data are supplied by the U.S Department of Energy and taken at Denver, Colorado[23]. Mars data are taken from the MCD corresponding to 0N 0E[24], and the Lunar equator temperatures are taken from NASA's Lunar Reconnaissance Orbiter (LRO) [25].

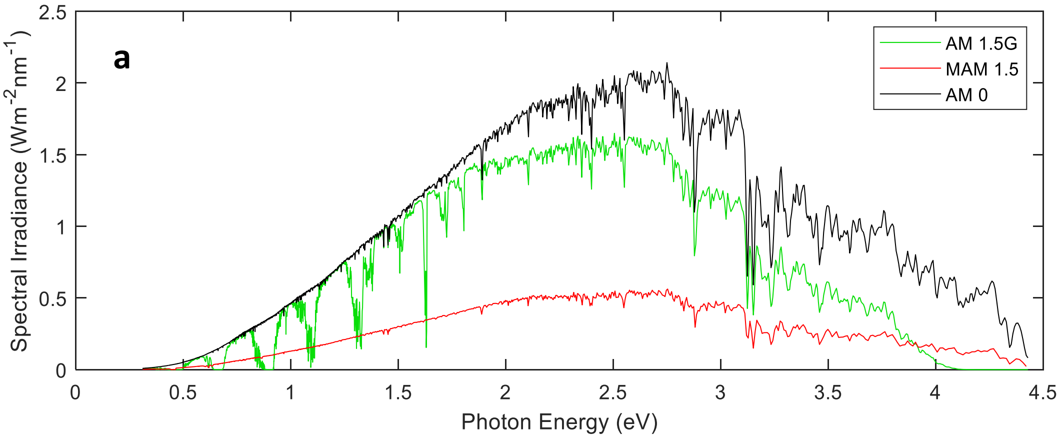

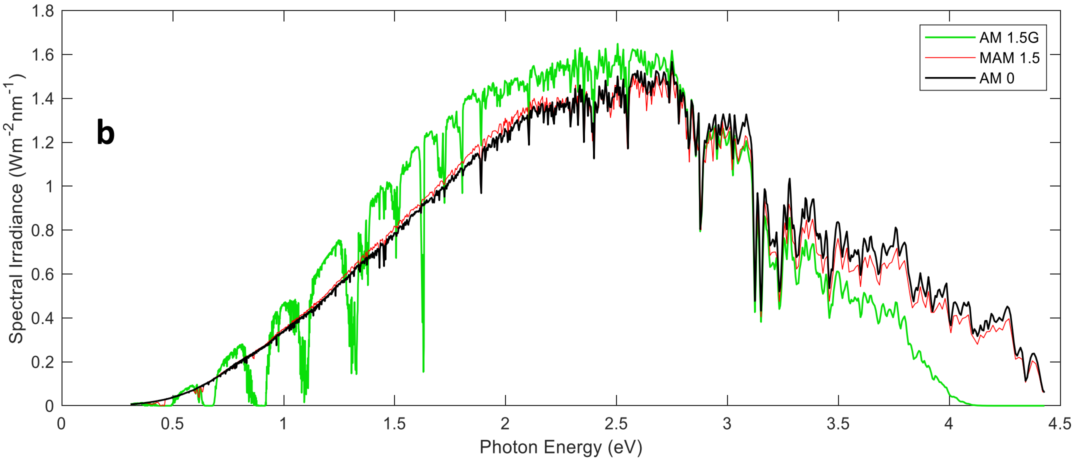

SI Figure 2 | Comparison of solar spectra used in models. a Spectral comparison to examine differences in photon energy distributions on Earth, Moon and Mars. **b** Spectral comparison where integrated power density of AM 0 and MAM 1.5 has been normalised to 1000 Wm^{-2} (AM 1.5 G) to examine relative differences in photon energy distributions.

IV Model Validations

SI Table 3 contrasts our PEC water-splitting results where applicable with Fountaine *et al.,* who reports comparable water splitting efficiencies under different terrestrial conditions. A significant correlation of our terrestrially derived efficiencies and ideal bandgap combinations can be observed when results are compared to Fountaine *et al.,* who provides three distinct scenarios for their PEC models. The divergences from our reported, high-performance realistic case can mainly be attributed to the applied catalytic exchange current (i_θ) values from Earth abundant OER catalysts. Fountaine *et al.* predict higher ε_g requirements because they are modelling an inherently less effective electrocatalyst in their real 2 case. We also employ series R_s and shunt R_{sh} resistance terms solved iteratively and compare them to the normalised resistance terms of Fountaine *et al.,* who also only used them in their real 2 case. Our models were run with the aid of Warwick's Scientific Computing Research Technology Platform (SCRTP) to facilitate the high computing resources needed for accurate multi-junction models looping through thousands of ε_g per photoabsorber combinations whilst using absolute series (R_s) and shunt (R_{sh}) resistance terms.

Our CO model is validated by previous work of Blake *et al*. We also overlay experimental data points by Verma *et al.* in SI Figure 6. The CH_4 model contains overlaid experimental data validated by the experimental work of Rasouli *et al.* Validation against current literature proved problematic for our MAM owing to the scarcity of extra-terrestrial solar irradiance spectra. Of the limited available comparisons, two spectra did provide a significant correlation with our results[26,27]. Differences between the intensity of the spectra can be attributed to the different solar zenith angle used. We have chosen to display several variations of our MAM to highlight the strong dependence of the spectral intensity on solar zenith angle variations.

Model	This article	Fountaine *et al.* [2]
Ideal case SJ Earth	30.7%, 1.59 eV	30.6 %, 1.59 eV
Ideal case SJ Moon	28.4%, 1.59 eV	-
Realistic case SJ Earth	11.0%, 2.07 eV	5.4%, 2.53 eV (real 2)<br>15.1%, 2.05 eV (real 1)
Realistic case SJ Moon	10.2%, 2.14 eV	-
Ideal case DJ Earth	40.3%, 0.51 eV, 1.39 eV	40.0%, 0.52 eV, 1.40 eV
Ideal case DJ Moon	38.1%, 0.57 eV, 1.32 eV	-
Realistic case DJ Earth	20.2%, 1.05 eV, 1.69 eV	16.2%, 1.38 eV, 1.93 eV (real 2)<br>28.3%, 0.92 eV, 1.59 eV (real 1)
Realistic case DJ Moon	17.9%, 1.08 eV, 1.73 eV	-
Ideal case TJ Earth	28.1%, 0.31 eV, 1.04 eV, 1.68 eV	28.3%, ε_g not specified
Ideal case TJ Moon	29.4%, 0.31 eV, 0.90 eV, 1.58 eV	-
Realistic case TJ Earth	15.9%, 0.79 eV, 1.27 eV, 1.84 eV	17.3%, 0.93 eV, 1.36 eV, 1.91 eV (real 2)<br>25.4%, ε_g not specified (real 1)
Realistic case TJ Moon	14.6%, 0.83 eV, 1.26 eV, 1.88 eV	-

SI Table 3 | Summary of PEC model outputs presented here and obtained by Fountaine *et al*. Here, the bandgap (ε_g) combinations are given alongside the solar-to-hydrogen conversion efficiency (STH, in %). SJ stands for singe-junction, DJ for dual-junction and TJ for triple-junction.

V Solar Fuel Production Model

The volume of solar fuel produced was calculated using Faraday's law of electrolysis for each quadrant of local time. For Earth and Mars, this corresponds to 1/24 of a full local day or sol, while for the Moon this equals to 1/24 of a lunar month. For the thermodynamically limiting case, we assume FE to be 100%, for the realistic cases the device operating current (i_{op}) is multiplied by the FE to give the partial current density of each device (j_x).

$$Production_{H_2} \qquad 2H^+ + 2e^- \rightarrow H_2 \tag{53}$$

$$Production_{CO} \qquad CO_2 + H_2O + 2e^- \rightarrow CO + OH^- \ (CO_2R) \tag{54}$$

$$Production_{CH_4} \qquad CO_2 + 8H^+ + 8e^- \rightarrow CH_4 + 2H_2O \ (CO_2R) \tag{55}$$

$$Volume\ produced\ per\ 365\ Earth\ days: \left(\sum_{t_{local}=1}^{24} \frac{j_x}{n_{e^-} \cdot F \cdot t_x} \cdot 24\ dm^3\right) \tag{56}$$

Here, t_x is the number of seconds in 1/24 of 365 Earth days ($1.31 \cdot 10^6$ s).

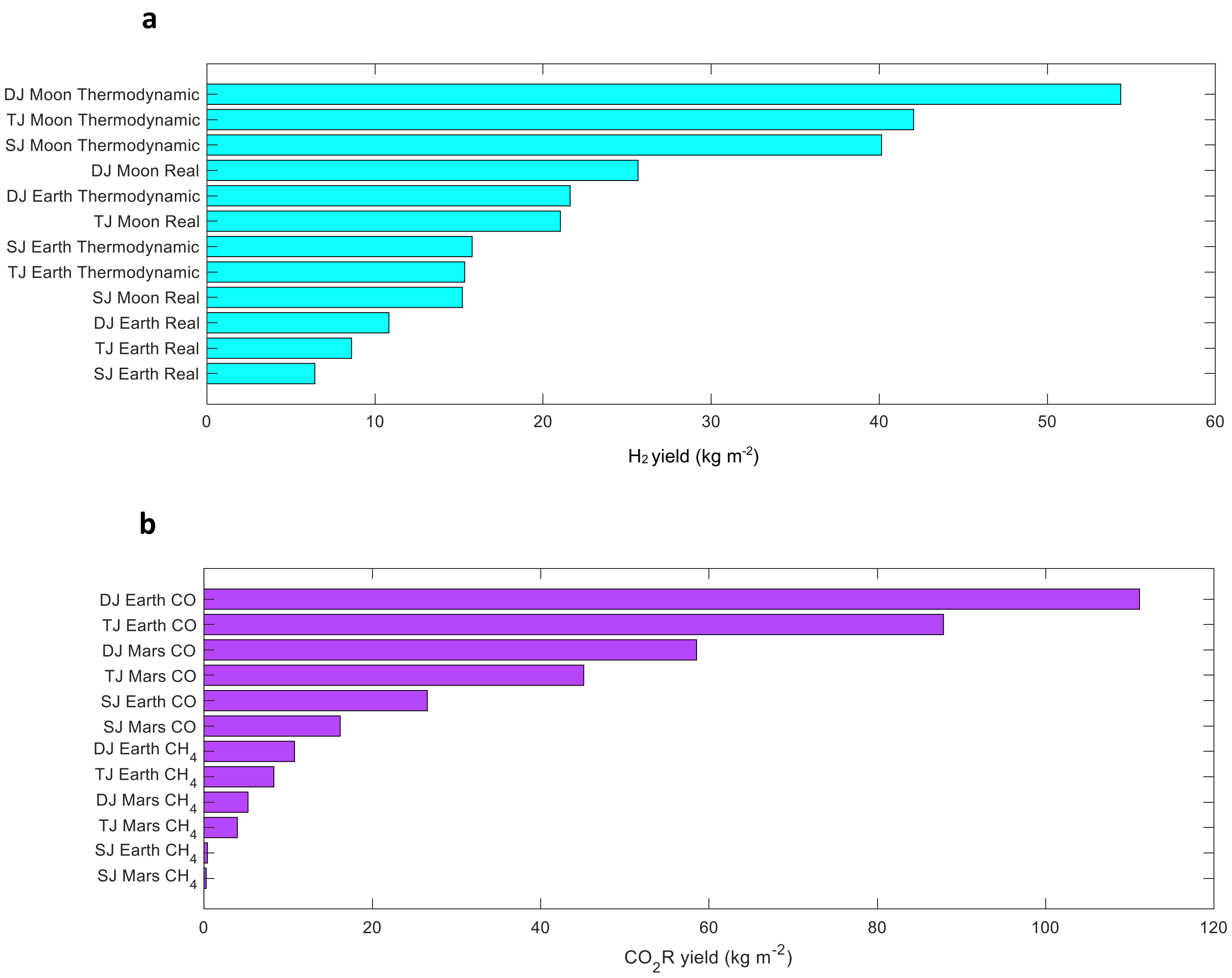

SI Figure 3 | Annual solar fuel production volumes. a Annual H_2 production by solar water-splitting. **b** Annual CO_2R production yielding CO and CH_4. The yield is given per device photoabsorber area.

VI H-Cell-type CO_2R and COR Solubility-Limited Current Density

To calculate the theoretical mass-transport limiting current density of the CO_2R in the H-cell configuration, we can employ Fick's law of diffusion, and assume that the bulk concentration is negligible at the interface[28]:

$$i_L = \frac{nFD_\theta[bulk]}{\delta} \tag{57}$$

Here, i_L is the limiting current density, n is the number of electrons transferred, F is Faraday's constant, D_θ is the diffusivity, $[bulk]$ is the reactant bulk concentration, and δ the diffusion layer thickness existing between the bulk concentration and electrode surface[28,29].

Parameter	Value	Ref.
n	2	-
D_{CO}	$2.03 \cdot 10^{-5}$ cm^2 s	30
D_{CO_2}	$1.92 \cdot 10^{-5}$ cm^2 s	30
$[CO]_{Bulk}$	0.99 mM	-
$[CO_2]_{Bulk}$	33 mM	-
δ	0 - 200 μM	28

SI Table 4 | Summary of parameters used for the limiting PEC current density calculations.

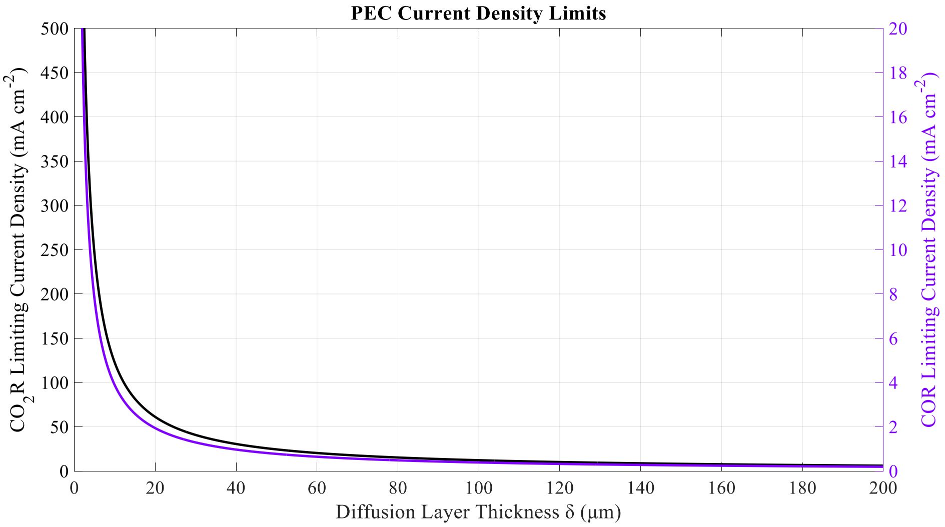

SI Figure 4 | Diffusion limited current density graph. Illustrating the difference in limiting current density of H-cells (δ ~ 100 μm) and GDE flow cells (δ ~ 100 nm). 33 mM is used as the solubility of carbon dioxide at standard conditions (298.15 K and 1 atm).

VII GDE-PV Design

Parameter	Value	Unit	Ref.
n_{e^-}	4	-	-
$i_{\theta\, OER}$	$2.50 \cdot 10^{-5}$	A m^{-2}	[19]
α_{OER}	0.50	-	-
U	1.229	V	-

SI Table 5 | Parameters used in calculating the GDE anodic overpotentials.

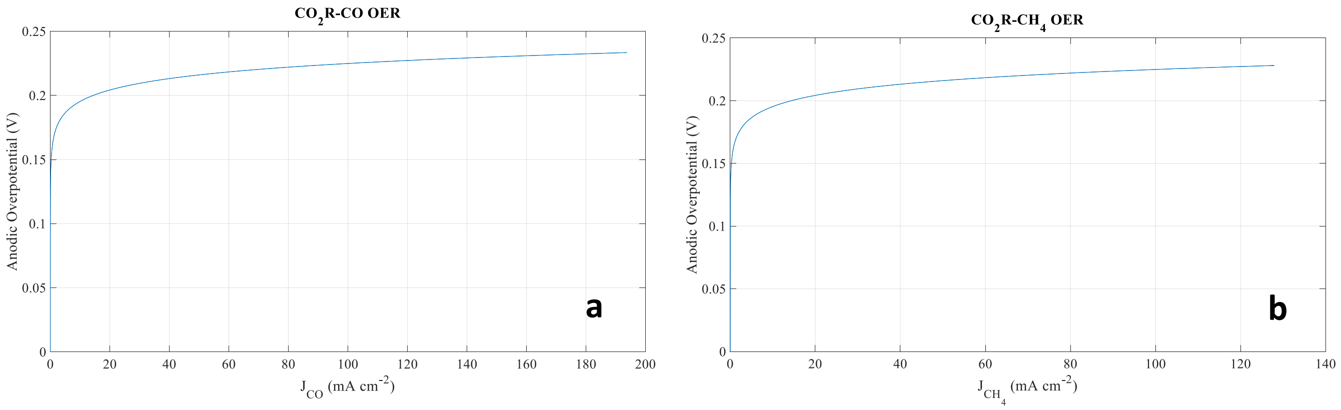

SI Figure 5 | CO_2R - OER overpotentials. The OER overpotential as a function of partial current density (J_{CO_2R}), which is added to the GDE cathode to calculate the cell potential required to drive the complete device for a CO and b CH_4 production.

The overpotential of the GDE-OER device was calculated using the Butler-Volmer equation and then added to the required cathode potential to determine the overall cell potential:

$$\eta_{(OER)} = \frac{RT}{4\alpha F} sinh^{-1}\left(\frac{i_{PV}}{2 \cdot i_{\theta\ PC}}\right) \tag{58}$$

$$\eta_{(cell)} = \left|\eta_{(cathode)}\right| + \left|\eta_{(OER)}\right| \tag{59}$$

The cathodic GDE models were recorded to serve as the electrochemical load curve needed to perform unassisted CO_2R on Earth and Mars. Through the previously outlined eq. (1) - (13) in the PEC modelling section, we generate the PV output curve and then solve for the point of intersection between the PV and electrochemical load curves to obtain the device operating current using polyxpoly (MATLAB).

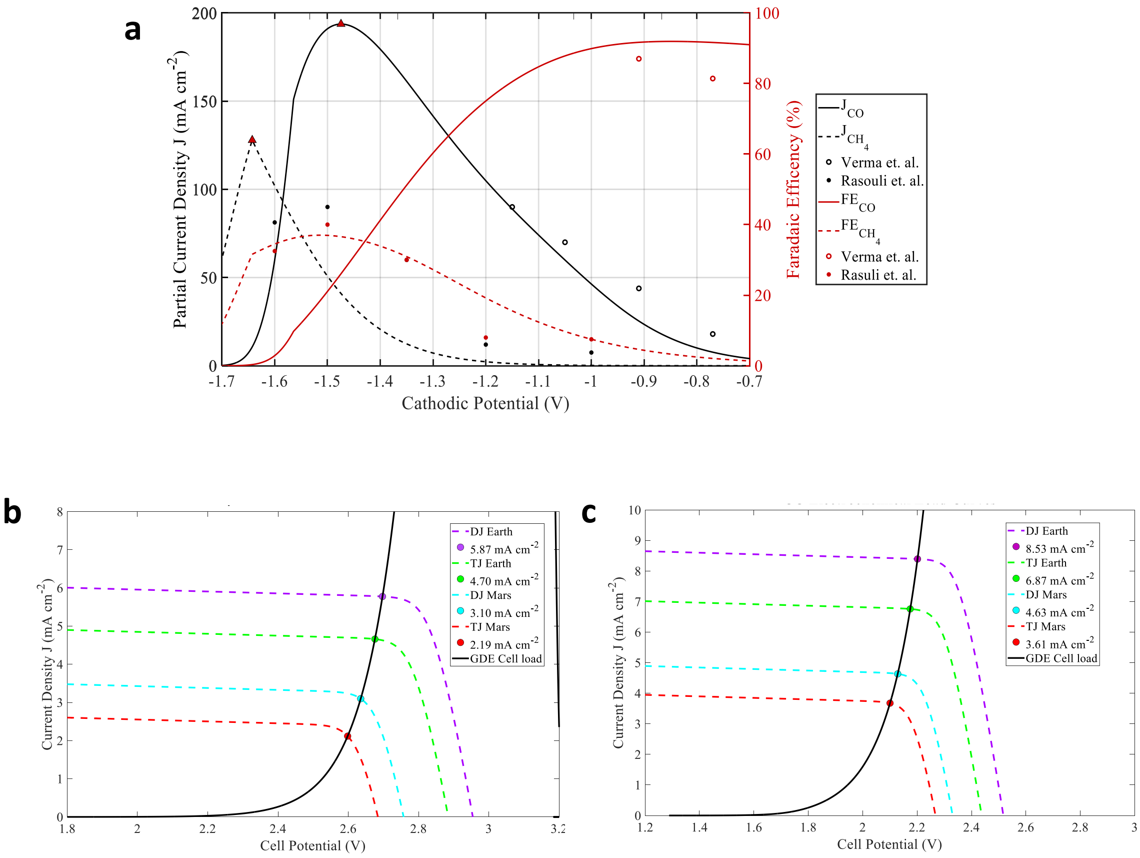

SI Figure 6 | Partial current density and Faradaic efficiency for both, CO and CH_4 models, as a function of potential and operating currents. a Experimental data points are overlaid with cathodic potentials (given *vs* RHE) to validate our theoretical models. **b** Realistic high-performance electrochemical load curves for CH_4 - producing tandem devices. **c** Realistic high-performance electrochemical load curves for CO - producing tandem devices. The intersection between the PV output curve and electrolyser load indicates the operating current (i_{op}) of the device.

VIII Lunar and Martian Soil Dust Performance Analysis

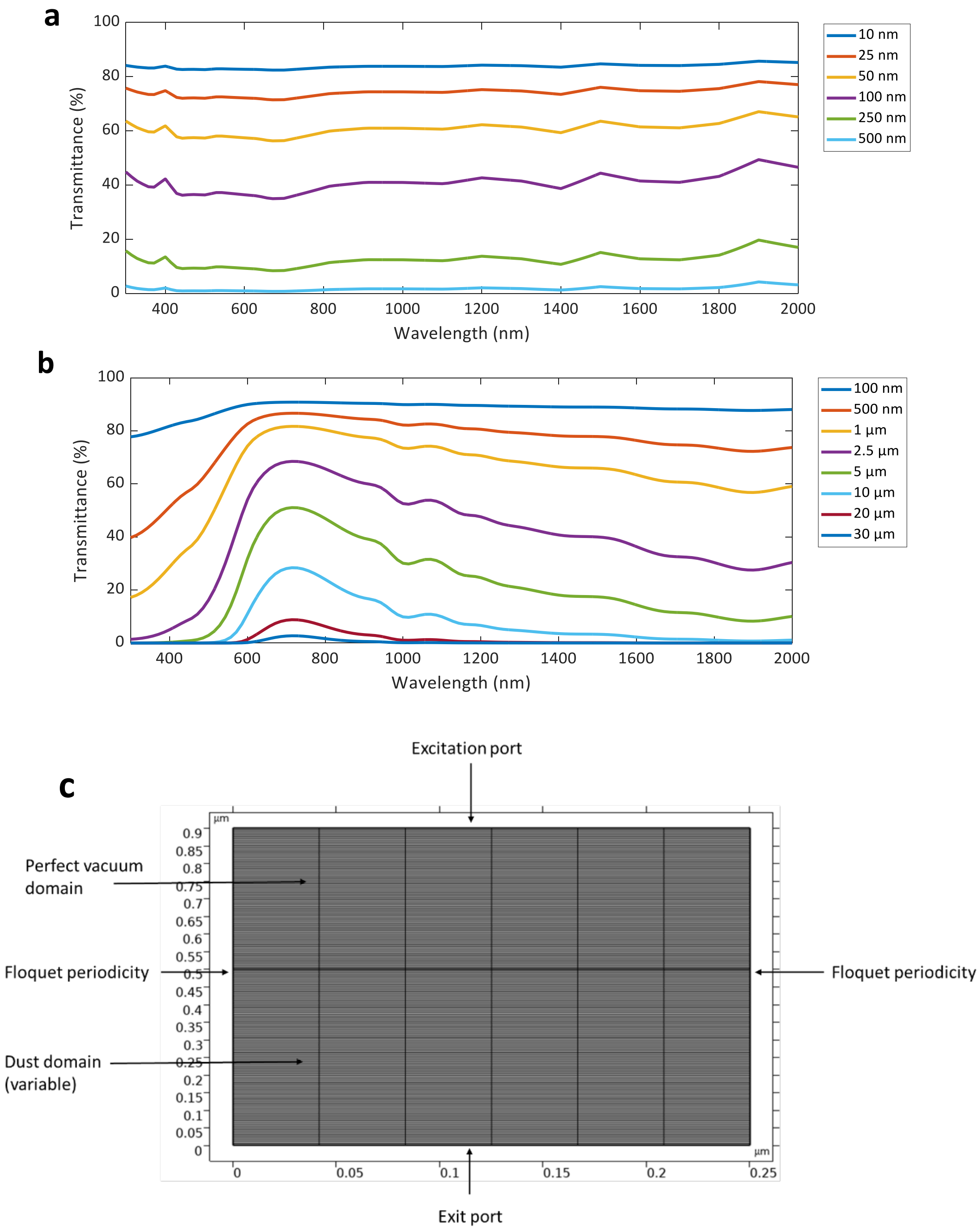

SI Figure 7 | TE and TM averaged COMSOL electromagnetic wave transmission model. a Transverse electric (TE) and transverse magnetic (TM) averaged electromagnetic (EM) transmission model based on the approximated lunar regolith dust complex refractive index. **b** Transverse electric (TE) and transverse magnetic (TM) averaged electromagnetic (EM) transmission model based on the Martian regolith dust complex refractive index[33]. **c** COMSOL model geometry, indicating boundary and domain conditions.

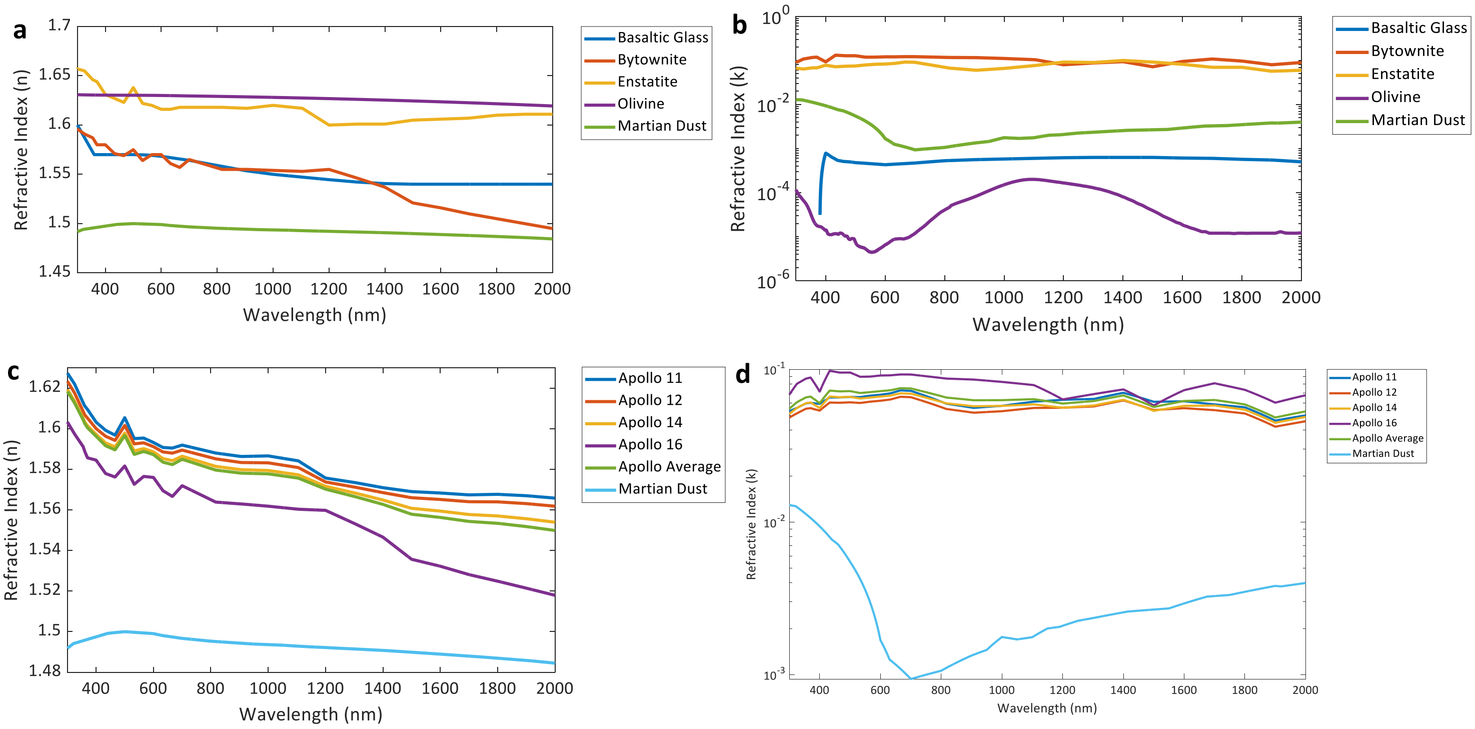

SI Figure 8 | Complex refractive indices of lunar and Martian soil dust. a Real component of complex refractive index (n) of major lunar regolith components[30-32] and Martian regolith dust[33]. **b** Imaginary component of the complex refractive index (k) of major lunar regolith components[30-32] and Martian regolith dust[33]. **c** Weighted real component of the complex refractive index (n) according to a composition analysis of several Apollo landings[34,35] compared to the one of Martian regolith dust[33]. **d** Weighted imaginary component of complex refractive index (k) according to composition analysis of lunar regolith from several Apollo landings[34,35] compared to the one of Martian regolith dust[33].

Material	Chosen complex refractive index	Ref.	Apollo 11 (wt %)	Apollo 12 (wt %)	Apollo 14 (wt %)	Apollo 16 (wt %)	Apollo average (wt %)
Plagioclase feldspar	Bytownite	30	21.4	23.2	31.8	69.1	36.4
Pyroxene	Enstatite	30	44.9	38.2	31.9	8.5	30.9
Olivine	Olivine	31	2.1	5.4	6.7	3.9	4.5
Mare glass	Basaltic glass	32	16.0	15.1	2.6	0.9	8.7
Highland glass	Basaltic glass	32	8.3	14.2	25.0	17.1	16.2
-	-	-	**92.7**	**96.1**	**98.0**	**99.5**	**96.6**

SI Table 6 | Components of weighted lunar regolith refractive index calculations. Lunar regolith sample mixtures from Apollo missions[34,35] were used to weight individual major regolith components to compute a first approximation of the wavelength dependent lunar regolith complex refractive index.

Model	Top semiconductor	Middle Semiconductor	Bottom semiconductor
Realistic case SJ (CO Mars)	≤488 nm = 67.4% attenuation	-	-
Realistic case SJ (CH_4 Mars)	≤420 nm = 74.0% attenuation	-	-
Realistic case DJ (CO Mars)	≤653 nm = 48.0% attenuation	-	>653 nm & ≤946 nm = 16.2% attenuation
Realistic case DJ (CH_4 Mars)	≤585 nm = 55.9% attenuation	-	>585 nm & ≤775 nm = 16.9% attenuation
Realistic case TJ (CO Mars)	≤602 nm = 53.7% attenuation	>602 nm & ≤827 nm = 16.1% attenuation	>827 nm & ≤1117 nm = 20.4% attenuation
Realistic case TJ (CH_4 Mars)	≤549 nm = 60.6% attenuation	>549 nm & ≤700 nm = 21.5% attenuation	>700 nm & ≤873 nm = 15.7% attenuation

SI Table 7 | Lunar and Martian regolith dust deposition and wavelength dependent performance impact on the light absorption of the semiconductor device.

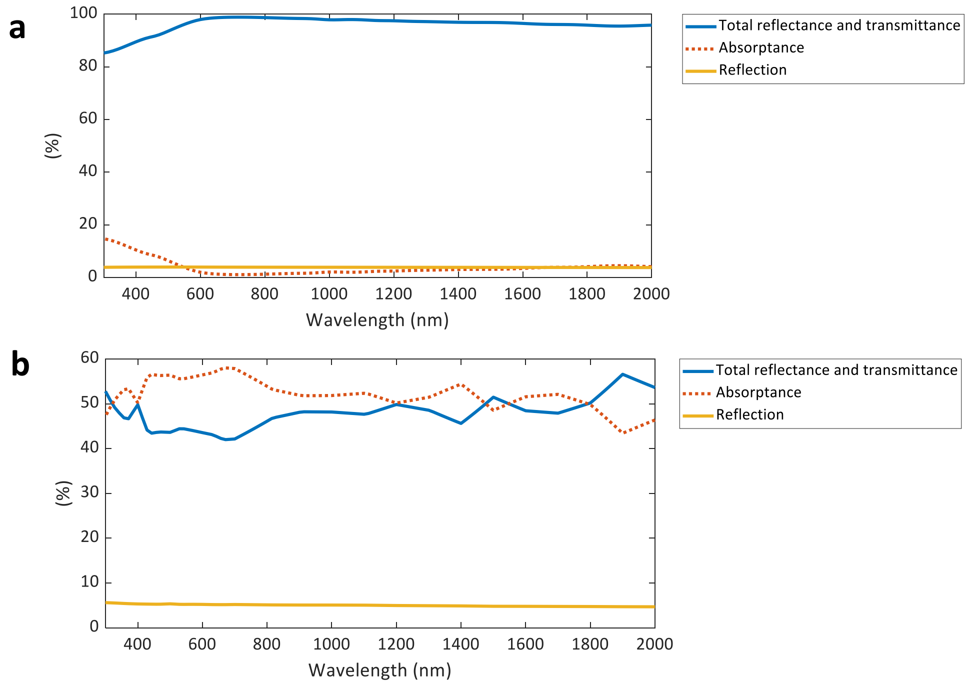

SI Figure 9 | COMSOL 100 nm layer dust deposition TM wave model. a Reflection, transmittance and absorbance effect of a 100 nm layer of Martian regolith dust layer under MAM 1.5 radiation. **b** Reflection, transmittance and absorbance effect of a 100 nm layer of lunar regolith dust layer under AM 0 radiation.

IX Natural Resource Abundances

Catalyst materials (ppm)						
**Material**	**Earth**	**Ref.**	**Moon**	**Ref.**	**Mars**	**Ref.**
Pt	$5 \cdot 10^{-3}$	37	6.9	38	$9.6 \cdot 10^{-3}$	39
Cu	44.7	37,40,41	8.2	38,42	2.6	39
Ru	$\leq 1 \cdot 10^{-3}$	36	4.9	38	$6.2 \cdot 10^{-3}$	39
Rh	$\leq 1 \cdot 10^{-3}$	36	1.1	38	$2.1 \cdot 10^{-3}$	39
Ir	$\leq 1 \cdot 10^{-3}$	36	4.8	38,43,44	$3.7 \cdot 10^{-3}$	39
Ni	46	40	$1.6 \cdot 10^{2}$	42-45	$3.6 \cdot 10^{2}$	39
Co	$3.2 \cdot 10^{2}$	38,40,41	95.7	39,42,44	96	39
Mo	$8.5 \cdot 10^{-2}$	40,41	9.8	38	0.5	39
Fe	$5.1 \cdot 10^{4}$	37,41	$7.9 \cdot 10^{4}$	42,45	$1.1 \cdot 10^{5}$	39
**Semiconductor materials (ppm)**						
Pb	15	37,41	$5.5 \cdot 10^{-5}$	38	0.3	39
S	$4.1 \cdot 10^{2}$	37,41	0.7	42	$3.6 \cdot 10^{2}$	39
Ge	1.4	40,41	0.6	38,43,46	0.6	39
Si	$2.9 \cdot 10^{5}$	37,41	$2.0 \cdot 10^{5}$	42,45	$2.13 \cdot 10^{5}$	39
Ga	18.8	40,41	3.4	38,42,46	8.7	39
As	3.7	40,41	0.9	38	$3.0 \cdot 10^{-2}$	39
O	$4.7 \cdot 10^{5}$	37,41	$4.3 \cdot 10^{5}$	42,45	$4.32 \cdot 10^{5}$	39
In	0.16	40,41	$7.5 \cdot 10^{-5}$	38,42,43,46	$1.0\ 10^{-2}$	39
P	$9.9 \cdot 10^{2}$	37,41	$2.5 \cdot 10^{2}$	38,44,45	$7.4 \cdot 10^{2}$	39

SI Table 8 | Natural abundances of elements of interest when constructing photoelectrochemical devices. Data shown are averaged from the indicated references.

Semiconductor	ε_g (eV)	Ref.
PbS	0.36	[47]
InAs	0.36	[47]
Ge	0.67	[47]
GaSb	0.72	[47]
Si	1.10	[47]
InP	1.30	[48]
GaAs	1.43	[47,48]
CdTe	1.50	[48]
CdSe	1.70	[49]
Cu_2O	2.00	[49]
Fe_2O_3	2.10	[49]
GaP	2.26	[50]
CdS	2.40	[49]
WO_3	2.60	[49]
Bi_2WO_3	2.80	[49]
SiC	3.00	[49]
TiO_2 rutile	3.00	[49]
ZnO	3.20	[49]

SI Table 9 | Summary of intrinsic semiconductor materials that have favourable band alignments for use in PEC water-splitting and CO_2R.

X References

1. Shockley, W. & Queisser, H. J. Detailed Balance Limit of Efficiency of p-n Junction Solar Cells. *J. Appl. Phys.* **32**, 510-519 (1961).

2. Fountaine, K. T., Lewerenz, H. J. & Atwater, H. A. Efficiency limits for photoelectrochemical water-splitting. *Nat. Commun.* **7**, 13706 (2016).

3. Xia, D., Wilkins, M. M. & Krich, J. J. External Radiative Efficiency In Multijunction Devices. In *47th IEEE Photovoltaic Specialists Conference (PVSC). 0549-0553* (2020).

4. deQuilettes, D. *et al.* Maximizing the external radiative efficiency of hybrid perovskite solar cells. *Pure Appl. Chem.* **92**, aop (2020).

5. Haussener, S., Gaudy, Y. & Tembhurne, S. *Photoelectrochemical Water Splitting: Theory, Experiment and Systems Analysis*. (The Royal Society of Chemistry, 2018).

6. Gutierrez, R. R. & Haussener, S. Modeling of Concurrent CO_2 and Water Splitting by Practical Photoelectrochemical Devices. *J. Electrochem. Soc.* **163**, H1008-H1018 (2016).

7. Kas, R. *et al*. Electrochemical CO_2 reduction on Cu_2O-derived copper nanoparticles: controlling the catalytic selectivity of hydrocarbons. *Phys. Chem. Chem. Phys.* **16**, 12194-12201 (2014).

8. Kweon, D. H. *et al.* Ruthenium anchored on carbon nanotube electrocatalyst for hydrogen production with enhanced Faradaic efficiency. *Nat. Commun.* **11**, 1278 (2020).

9. Jiang, R. *et al.* Ru@RuO_2 Core-Shell Nanorods: A Highly Active and Stable Bifunctional Catalyst for Oxygen Evolution and Hydrogen Evolution Reactions. *Energy Environ. Mat.* **2**, 201-208 (2019).

10. Marković, N. M., Grgur, B. N. & Ross, P. N. Temperature-Dependent Hydrogen Electrochemistry on Platinum Low-Index Single-Crystal Surfaces in Acid Solutions. *J. Phys. Chem. B* **101**, 5405-5413 (1997).

11. Blouin, M. & Guay, D. Activation of Ruthenium Oxide, Iridium Oxide, and Mixed RuxIr1B x Oxide Electrodes during Cathodic Polarization and Hydrogen Evolution. *J. Electrochem. Soc.* **144**, 573-581 (1997).

12. Tijani, A. S., Kamarudin, N. A. B. & Mazlan, F. A. B. Investigation of the effect of charge transfer coefficient (CTC) on the operating voltage of polymer electrolyte membrane (PEM) electrolyzer. *Int. J. Hydrog. Energy* **43**, 9119-9132 (2018).

13. Blake, J. W., Padding, J. T. & Haverkort, J. W. Analytical modelling of CO_2 reduction in gas-diffusion electrode catalyst layers. *Electrochim. Acta* **393**, 138987 (2021).

14. Rasouli, A. S. *et al.* CO_2 Electroreduction to Methane at Production Rates Exceeding 100 mA cm^{-2}. *ACS Sustain. Chem. Eng.* **8**, 14668-14673 (2020).

15. Hatsukade, T. *et al*. Insights into the electrocatalytic reduction of CO_2 on metallic silver surfaces. *Phys. Chem. Chem. Phys.* **16**, 13814-13819 (2014).

16. Pegis, M. L. *et al*. Standard Reduction Potentials for Oxygen and Carbon Dioxide Couples in Acetonitrile and *N*,N-Dimethylformamide. *Inorg. Chem.* **54**, 11883-11888 (2015).

17. Whipple, D. T., Finke, E. C. & Kenis, P. J. A. Microfluidic Reactor for the Electrochemical Reduction of Carbon Dioxide: The Effect of pH. *Electrochem. Solid-State Lett.* **13**, B109 (2010).

18. Newman, J. & Thomas-Alyea, K. E. *Electrochemical Systems*. (Wiley, 2012).

19. Zhou, L. Q. *et al.* A high-performance oxygen evolution catalyst in neutral-pH for sunlight-driven CO_2 reduction. *Nat. Commun.* **10**, 4081 (2019).

20. Weisenberger, S. & Schumpe, A. Estimation of gas solubilities in salt solutions at temperatures from 273 K to 363 K. *AIChE J.* **42**, 298–300 (1996).

21. Zwart, S. P. & McMillan, S. *Astrophysical Recipes*. (IOP Publishing, 2018).

22. Emde, C. *et al.* The libRadtran software package for radiative transfer calculations (version 2.0.1). *Geosci. Model Dev.* **9**, 1647-1672 (2016).

23. Sengupta, M. *et al*. The National Solar Radiation Data Base (NSRDB). *Renew. Sustain. Energy Rev*. **89,** 51-60 (2018).

24. Millour, E. *et al.* The Mars Climate Database (version 5.3). In *19th EGU General Assembly*. #12247 (2017).

25. National Aeronautics and Space Administration. Lunar Reconnaissance Orbiter. *PDS Geoscience Node* https://pds-geosciences.wustl.edu/missions/lro/default.htm (2022).

26. Deo, S., Kalchgruber, R. & Mayer, B. Radiative Transfer Calculations for the Atmosphere of Mars in the 200-900 nm Range. In *36th Annual Lunar and Planetary Science Conference. #1029* (2005).

27. Abel, A. J. *et al*. Photovoltaics-Driven Power Production Can Support Human Exploration on Mars. *Front. Astron. Space Sci.* **9**, 868519 (2022).

28. Jinli, Q., Yuyu, L. & Jiujun, Z. *Electrode Kinetics of CO Electroreduction.* (CRC Press, 2016).

29. Xing, Z. *et al*. Enhancing carbon dioxide gas-diffusion electrolysis by creating a hydrophobic catalyst microenvironment. *Nat. Commun.* **12**, 136 (2021).

30. Egan, W. G. & Hilgeman, T. W. *Optical Properties of Inhomogeneous Materials*. (Academic Press, 1979).

31. Fabian, D. *et al*. Steps toward interstellar silicate mineralogy. *A&A.* **378**, 228-238 (2001).

32. Pollack, J. B., Toon, O. B. & Khare, B. N. Optical properties of some terrestrial rocks and glasses. *Icarus* **19**, 372-389 (1973).

33. Wolff, M. J. *et al.* Wavelength dependence of dust aerosol single scattering albedo as observed by the Compact Reconnaissance Imaging Spectrometer. *J. Geophys. Res.* **114**, (2009).

34. Papike, J. J., Taylor, L. & Simon, S. *Lunar Minerals*. (Cambridge University Press, 2012).

35. Papike, J. J., Simon, S. B. & Laul, J. C. The lunar regolith: Chemistry, mineralogy, and petrology. *Rev. Geophys.* **20**, 761 (1982).

36. Worstell, J. H. *Diffusion: Mass Transfer in Fluid Systems.* (Cambridge University Press, 2009).

37. Rumble, J. R. *CRC handbook of chemistry and physics.* (CRC Press, 2021).

38. Ganapathy, R. & Anders, E. Bulk composition of the moon and earth, estimated from meteorites. In 5th *Lunar and Planetary Science Conference.* 1181-1206 (1974).

39. Yoshizaki, T. & McDonough, W. F. The composition of Mars. *Geochim. Cosmochim. Acta* **273**, 137-162 (2020).

40. Hu, Z. & Gao, S. Upper crustal abundances of trace elements: A revision and update. *Chem. Geol.* **253**, 205-221 (2008).

41. Yaroshevsky, A. A. Abundances of chemical elements in the Earth's crust. *Geochem. Int.* **44**, 48-55 (2006).

42. Wänke, H*. et al.* Major and trace elements in lunar material. In *Apollo 11 Lunar Science Conference*. 1719-1727 (1970).

43. Wolf, R. & Anders, E. Moon and Earth: compositional differences inferred from siderophiles, volatiles, and alkalis in basalts. *Geochim. Cosmochim. Acta* **44**, 2111-2124 (1980).

44. Lawrence, D. J. *et al*. Mapping the elemental composition of the moon: Current results of the Lunar Prospector gamma ray spectrometer. In *Meteoritical Society Meeting.* #980770 (1998).

45. Prettyman, T. H*. et al.* Elemental composition of the lunar surface: Analysis of gamma ray spectroscopy data from Lunar Prospector. *J. Geophys. Res. Planets* **111**, E12007 (2006).

46. Baedecker, P. A. & Wasson, J. T. Gallium, Germanium, Indium, and Iridium in Lunar Samples. *Sci.* **167**, 503-505 (1970).

47. Dharmadasa, I., Ojo, A., Salim, H. & Dharmadasa, R. Next Generation Solar Cells Based on Graded Bandgap Device Structures Utilising Rod-Type Nano-Materials. *Energies* **8**, 5440-5458 (2015).

48. Jian, J. & Sun, J. A Review of Recent Progress on Silicon Carbide for Photoelectrochemical Water Splitting. *Solar RRL* **4**, 2000111 (2020).

49. Ong, W. J. *et al*. Graphitic Carbon Nitride (g-C3N4)-Based Photocatalysts for Artificial Photosynthesis and Environmental Remediation: Are We a Step Closer To Achieving Sustainability? *Chem. Rev.* **116**, 7159-7329 (2016).

50. Aparna, A. R., Brahmajirao, V. & Karthikeyan, T. V. Review on Synthesis and Characterization of Gallium Phosphide. *Procedia Mat. Sci.* **6**, 1650-1657 (2014).
